# Anatomical and Radiographic Study on the Skull and Mandible of the Common Opossum (*Didelphis Marsupialis* Linnaeus, 1758) in the Caribbean

**DOI:** 10.3390/vetsci5020044

**Published:** 2018-04-23

**Authors:** Reda Mohamed

**Affiliations:** 1 Department of Basic Veterinary Sciences, School of Veterinary Medicine, Faculty of Medical Sciences, The University of the West Indies, St. Augustine, Trinidad and Tobago; reda.mohamed@sta.uwi.edu; Tel.: +645-3232-4206; 2 Anatomy and Embryology Department, Faculty of Veterinary Medicine, Beni-Suef University, Beni-Suef 62511, Egypt

**Keywords:** anatomy, radiographic, skull, opossum, marsupial, Caribbean

## Abstract

Common opossums (*Didelphis marsupialis*) are found throughout the Caribbean island of Trinidad and Tobago. The present work was conducted on 10 skulls and mandibles of the common opossum to describe the osteology and foramina of these skulls and mandibles grossly and radiographically. The information that is garnered can be used to detect, diagnose, and treat head affections, as well as for comparative studies with the skulls and mandibles of other similar species. The skulls and mandibles were prepared and cleaned using standard method. All of the characteristic features of various standards views of the skulls bones, including dorsal, lateral, caudal and midsagittal, and the lateral and caudal views of the mandibles as well as the foramina of the skulls and mandibles were described and discussed. Each skull was divided into long facial and short cranial regions. No supraorbital foramen was observed in the skulls. The tympanic bulla was absent while there was the tympanic process of the alisphenoid. The temporal process of the zygomatic bone, zygomatic process of maxilla, and zygomatic process of the squamosal bone formed the zygomatic arch. The dental formula was confirmed. The bones and foramina of the skull and mandible were similar to other marsupial species and were homologue to that of other mammals.

## 1. Introduction

The genus Didelphis has six species of marsupials that are found only in the Americas, which are *Didelphis marsupialis*, *Didelphis aurita*, *Didelphis albiventris*, *Didelphis pernigra*, *Didelphis virginiana,* and *Didelphis imperfecta* [1]. Common opossums (*D*. *marsupialis*) are found throughout much of Central and South America and the Caribbean. They are characterized by a thick coat of fur, sharp claws, long whiskers, long tail, and black ring around both eyes as well as large black ears. The common opossum has an economic value as it is sold by hunters for meat in Trinidad and Tobago. The animal uses its head for deglutition and olfaction. The head of the animal has the vital organs, such as brain, eyes, ears, teeth, tongue, lips, and nose [2]. The skull gives the phylogenetic appearance of the head of animal species, such as the feline and the camel [3,4]. Moreover, the taxonomic affiliations can be detected via the skull morphology as mentioned in the camel and in the common shrew [4,5]. There was no description of the bones and foramina of the skull and mandible of the common opossum, except in the description, which was given in the white-eared opossum (*D*. *albiventris*) in Brazil [6], in the short-tailed opossum (*D*. *Marsupialis*) [7], and in the marsupial *Marmosa murina* [8]. Thus, the aim of this study is to give a detailed gross and radiographic description of the bones and foramina of the skull and mandible of the common opossum from the Caribbean island of Trinidad and Tobago. This information could be used in the identification of the skull and mandible of the common opossum, as well as to help the veterinarian to detect, diagnose, and treat the head affections in this species.

## 2. Materials and Methods

The skulls of ten (three males and seven females) adult common opossums were used. The opossums were obtained to use for research purposes in the School of Veterinary Medicine, Faculty of Medical Sciences, University of the West Indies, Trinidad and Tobago. The skulls were collected, examined and prepared. The skulls were free from any skeletal deformities. The skulls were cut at the occipitoatlantal joint. The skulls were boiled to remove the skin and muscles then left to dry for two weeks. The skulls were bleached in a sealed container for four days in 3% hydrogen peroxide, and then left to dry for two weeks [9]. The gross and radiographic images of the skull were taken in different views (dorsal, ventral, caudal, and midsagittal) for documentation using a digital camera and Siemens mobile full wave X-ray machine (Siemens Medical Solutions, Erlangen, Germany). The bones and foramina of the skull were named according to Schimming et al. [6], Wible [7], and Nomina Anatomica Veterinaria [10].

## 3. Results

The skull of the opossum has an elongated facial region and short basicranial region. The bones and foramina of the two regions are described and documented in different standard views.

### 3.1. The Bones of the Facial Region

**Figure 1:**
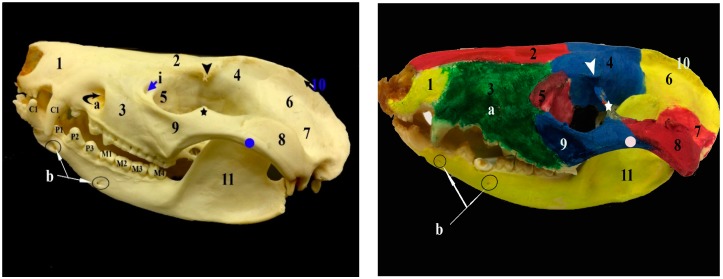
Photograph showing the lateral view of the skull and mandible of the common opossum. 1. premaxilla bone; 2. nasal bone; 3. maxilla bone; 4. frontal bone; 5. lacrimal bone; 6. parietal bone; 7. squamosal bone; 8. zygomatic process of squamosal bone; 9. zygomatic bone; 10. external sagittal crest; 11. mandible; arrow head: postorbital process of frontal bone; star: frontal process of zygomatic bone; circle: temporal process of zygomatic bone; a. infraorbital foramen; b. mental foramina; C1. canine teeth; P1–3. premolar teeth; and, M1–4. molar teeth.

**Figure 2:**
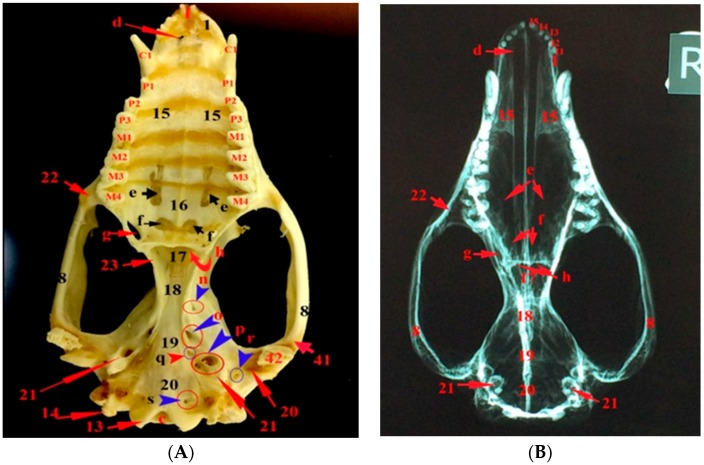
Images showing the gross anatomy (**A**) and radiography (**B**) of the ventral view of the skull of the common opossum. 1. premaxilla bone; 8. zygomatic process of squamosal bone; 14. jugular process; 15. palatine process of maxilla; 16. palatine bone; 17. vomer bone; 18. presphenoid bone; 19. basisphenoid bone; 20. basioccipital bone; 1. tympanic process of the alisphenoid; 22. zygomatic process of maxilla; 23. pterygoid bone; 41. prearticular process; 42. retroarticular process; c. foramen magnum; d. palatine fissure; e. maxillopalatine fenestra; palatine fenestra; g. caudal palatine foramen; h. choana; o. caudal alar foramen; p. carotic canal; q. transverse canal foramen; r. stylomastoid foramen; s. hypoglossal canal; I1–5. upper incisor teeth; C1. upper canine teeth; P1–3. upper premolar teeth; and, M1–4. upper molar teeth.

**Figure 3:**
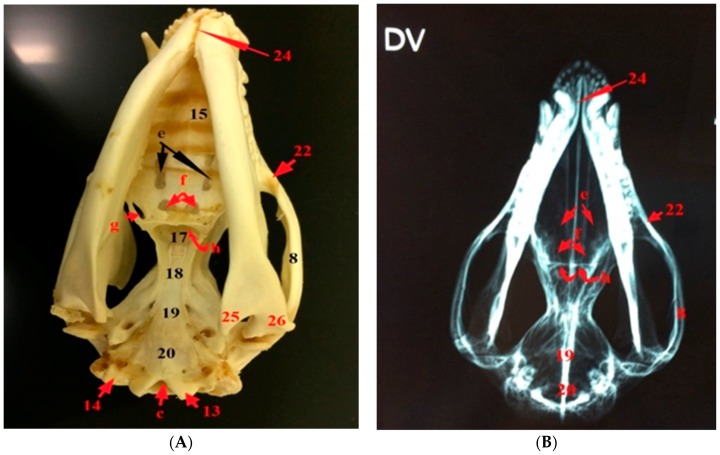
Images showing the gross anatomy (**A**) and radiography (**B**) of the dorsoventral view of the skull and mandible of the common opossum. 8. zygomatic process of squamosal bone; 13. occipital condyle; 14. jugular process; 15. palatine process of maxilla; 17. vomer bone; 18. presphenoid bone; 19. basisphenoid bone; 20. basioccipital bone; 22. zygomatic process of maxilla; 24. mandibular symphysis; 25. angular process; 26. condylar process c. foramen magnum; e. maxillopalatine fenestra; f. palatine fenestra; g. caudal palatine foramen; and, h. choana.

The bony part of the face is formed by the nasal, lacrimal, and zygomatic (jugal) bones in the dorsal and lateral aspects of the skull; premaxilla, maxilla in the dorsal, lateral, and ventral aspects; of the skull and pterygoid, ethmoturbinate, and vomer bones in the ventral aspect of the skull. Moreover, the choanal region and hard palate are seen in the ventral aspect of the skull (Figure 1, Figure 2 and Figure 3).

**Figure 4:**
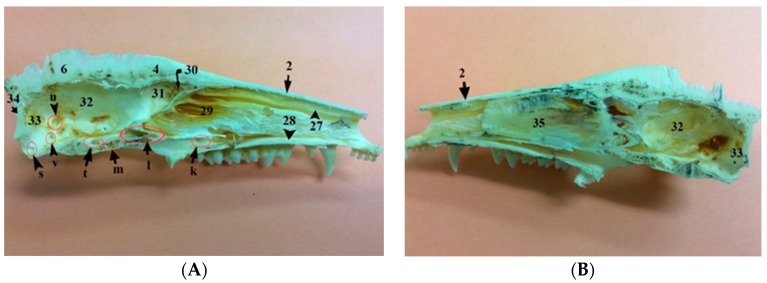
Photographs showing the sagittal section of the left (**A**) and right (**B**) halves of the skull of the common opossum. 2. nasal bone; 4. frontal bone; 6. parietal bone; 27. ethmoidal crest; 28. conchal crest; 29. ethmoturbinate; 30. cribriform plate of the ethmoid; 31. rostral cranial fossa; 32. middle cranial fossa; 33. caudal cranial fossa; 34. occipital bone; 35. perpendicular plate of the ethmoid; k. sphenopalatine foramen; l. orbital fissure; m. round foramen; s. hypoglossal canal; t. oval foramen; u. internal acoustic meatus; and, v. jugular foramen.

The hard palate is formed by the palatine bones as well as the palatine processes of the premaxilla and maxilla. The choanae are located at the terminal part of the hard palate. The midsagittal view of the face region of the skull shows the two halves of the nasal cavity separated from each other and from the cranium by the nasal septum and cribriform plate, respectively (Figure 4). Each half of the nasal cavity has ethmoturbinate dorsocaudally and ventral nasal concha ventrorostrally. The dorsal nasal concha protrudes medially from the conchal crest, while the ventral nasal concha medially protrudes from the ethmoidal crest. The vomer bone and perpendicular plate of the ethmoid bone forms the nasal septum, which has the septal cartilage rostrally.

The nasal bones are paired bones in the nasal cavity roof. Each nasal bone is notched to form the dorsal border of the external osseous nasal aperture rostrally. The nasal bones are attached from cranial to caudal with the premaxillae, maxillae and frontals. Neither cranial nasal notch nor foramina are present in the nasal bones. The premaxillae are paired bones that form the lateral and ventral walls of the nasal cavity. Each premaxilla bone houses the alveoli for the five upper incisors. The premaxilla bones are attached to the nasal bones dorsally and to the maxillae caudally. The maxilla bones form the wall of the snout and hard palate laterally and they house the upper canine, upper three premolars and upper four molars. The maxillae are attached to the premaxillae cranially, and with the nasal, frontal, lacrimal, and zygomatic bones caudally. The lacrimal bones form the cranial rim of the orbit. The zygomatic bones are the principal parts of the zygomatic arches. The zygomatic bones have the frontal processes of zygomatic bones. There are many processes that contribute to the formation of the zygomatic arch, such as the temporal process of the zygomatic bone and the zygomatic process of the maxilla, as well as the zygomatic processes of the squamosal and maxilla bones. The palatine bones are paired in the palate and orbit. They have a horizontal process and perpendicular processes that contribute to the hard palate, as well as the orbit and choanae, respectively. The pterygoid bones form the lateral wall and roof of the nasopharyngeal passage. The vomer bone is a thin elongated bone, which contributes to the nasal cavity cranially and articulates with the presphenoid, maxilla and premaxilla bones (Figure 1, Figure 2 and Figure 4).

### 3.2. The Foramina of the Facial Region

**Figure 5:**
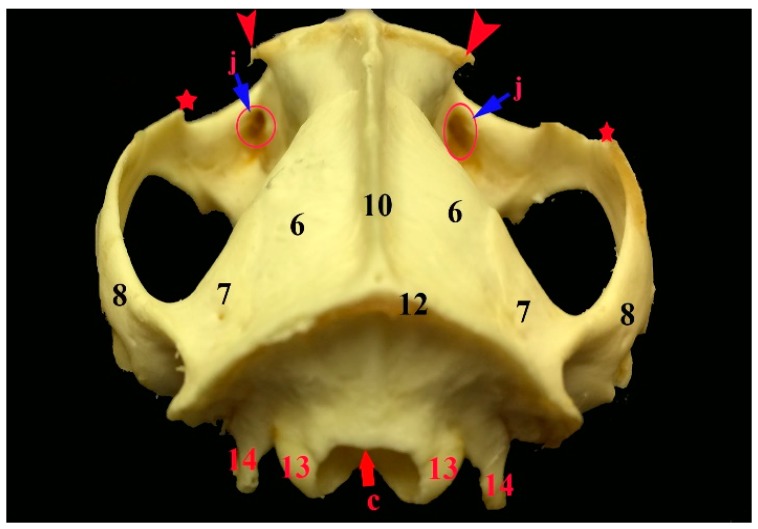
Photograph showing the nuchal surface of the occipital bone of the common opossum. 6. parietal bone; 7. squamosal bone; 8. zygomatic process of squamosal bone; 9. zygomatic bone; 10. external sagittal crest; 12. nuchal crest; 13. occipital condyle; 14. jugular process; arrow head: zygomatic process of frontal bone; star: frontal process of zygomatic bone; circle: temporal process of zygomatic bone; c. foramen magnum; j. maxillary foramen; k. sphenopalatine foramen; l. orbital fissure; and, m. round.

**Figure 6:**
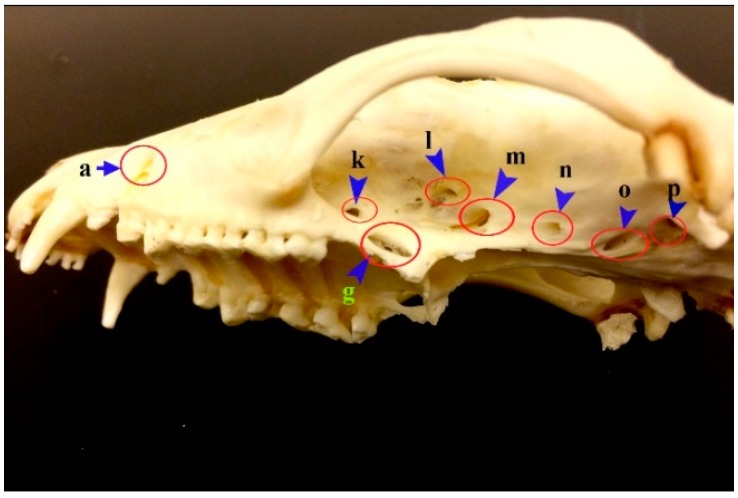
Photograph showing the foramina in the lateral aspect of the skull of the common opossum. a. infraorbital foramen; g. caudal palatine foramen; k. sphenopalatine foramen; l. orbital fissure; m. foramen rotundum; n. rostral alar foramen; o. caudal alar foramen; p. carotic canal; q. transverse canal foramen.

The foramina of the facial region (Figure 1, Figure 4, Figure 5 and Figure 6) of the skull of the common opossum are the infraorbital and lacrimal foramina dorsally and a rostral group of foramina, which is located in the pterygopalatine fossa and consists of the maxillary, sphenopalatine, and caudal palatine foramina. The ventral view of the skull shows the palatine fissures (incisive foramen), maxillopalatine fenestra (major palatine foramen), and palatine fenestra (minor palatine foramina). The infraorbital canals are formed by the infraorbital foramina cranially and maxillary foramina caudally. The lacrimal foramina are two foramina on each lacrimal bone. The incisive foramen is present within the maxilla on the cranial hard palate. The major palatine foramina are elongated craniocaudally. The palatine fenestrae are present caudaomedial to the last upper molar. The sphenopalatine foramina are located within the palatine near the maxillary foramina.

### 3.3. The Bones of the Basicranial Region

The bony part of the basicranial region of the skull of the common opossum consists of the frontal and parietal bones in the dorsal and lateral aspects of the skull; the squamosal and external sagittal crest in the dorsal aspect of the skull; the sphenoid bones, temporal, and pterygopalatine fossae in the lateral aspect of the skull; the nuchal crest, occipital condyles, and jugular processes in the caudal aspect of the skull and the base of the cranium, occipital, alisphenoid with its tympanic process, basioccipital; presphenoid, squamosal and petrosal bones, prearticular process, retroarticular process, and mandibular fossa in the ventral aspect of the skull. The midsagittal view of the bsicranial region of the skull shows the cranial cavity which has rostral cranial, middle cranial and caudal cranial fossae. The zygomatic arch is seen clearly in the lateral aspect of the skull (Figure 1, Figure 2, Figure 3, Figure 4 and Figure 5).

**Figure 7:**
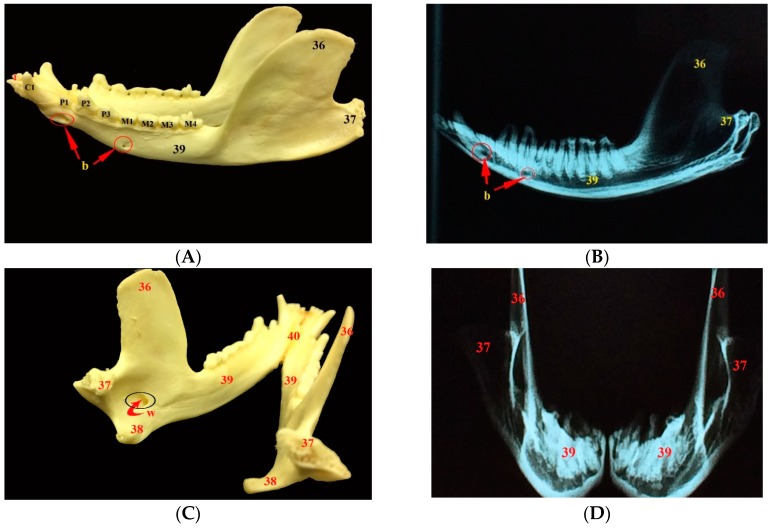
Images showing gross anatomy (**A**) and radiography (**B**) of the lateral view as well as gross anatomy (**C**) and radiography (**D**) of the caudal view of the mandible of the common opossum. 36. coronoid process; 37. condylar process; 38. angular process; 39. ramus of the mandible; 40. body of the mandible; 41. prearticular process; b. mental foramina; w. mandibular foramen; I. lower incisor teeth; C1. lower canine teeth; P1–3. lower premolar teeth; and, M1–4. lower molar teeth.

The frontal bone forms the roof of the skull and contributes to the orbital and temporal fossae medially. Cranially, the frontals attaches to the nasals at a roughly V-shaped suture and with the maxilla and the lacrimal by a narrow connection. The frontal bone has a zygomatic process. The parietal bones form the bulk of the roof of the skull caudally, and contributed to the caudal side wall of the braincase. The presphenoid bone is a single bone in the nasopharyngeal passage roof and it contributed to the nasal septum. It contacts with the palatine, pterygoid, and basisphenoid bones. The basisphenoid bone is a single bone occupying the midline of the brain stem and is situated between the presphenoid and basioccipital bones. The basioccipital bone is a single bone that is situated between the petrosal and foramen magnum and it forms the base of the skull. The alisphenoid bone is found on either side of the basisphenoid and fuses with it and it contributes to the base of the skull and side wall of the braincase. The squamosal bone contributes to the braincase, zygomatic arch, and glenoid fossa. The temporomandibular joint is found between the glenoid fossa of the skull and the coronoid process of the mandible. The rostral cranial fossa is situated caudal to the cribriform plate. The bony orbit is formed by the frontal, lacrimal bone, zygomatic bone, maxillary bone, palatine bone, and squamosal bone as well as the basisphenoid and alisphenoid of the sphenoid bone. The dental formula of the ten common opossums is 50 teeth; Incisor (I) 5/4, Canines (C) 1/1, Premolars (Pm) 3/3, Molars (M) 4/4 × 2 = 50 (Figure 1, Figure 2 and Figure 7).

### 3.4. The Foramina of the Basicranial Region

**Figure 8:**
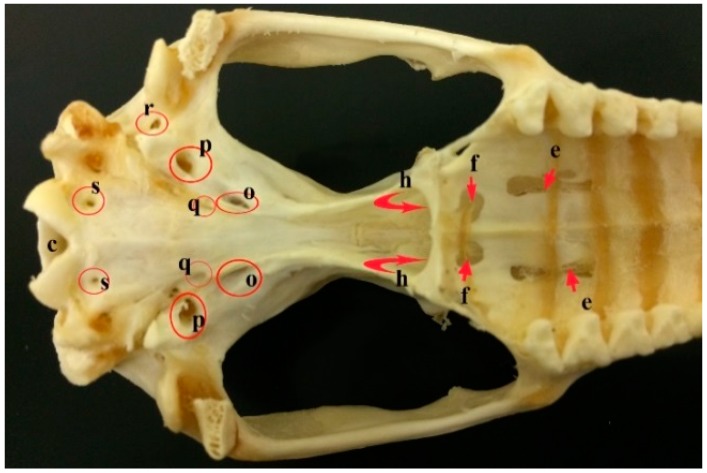
A photograph showing the foramina in the ventral aspect of the skull of the common opossum. c. foramen magnum; e. maxillopalatine fenestra; f. palatine fenestra; h. choanae; o. caudal alar foramen; p. carotic canal; q. transverse canal foramen; r. stylomastoid foramen; and, s. Hypoglossal canal.

The foramina of the cranial region of the skull of the opossum are the external acoustic meatus, the carotic canal, the minor palatine foramen, the stylomastoid foramen, the sphenorbital fissure, the foramen ovale, the foramen rotundum, the jugular foramen, the hypoglossal canal, the transverse canal foramen, the internal acoustic meatus, and the foramen magnum, as well as the rostral and the caudal foramina that formed the pterygoid canal (Figure 2, Figure 3, Figure 4, Figure 5 and Figure 6 and Figure 8).

### 3.5. The Mandible

The mandible is the largest bone of the skull. The left and right mandibles are fused rostrally at the mandibular symphysis, a fibrocartilaginous joint. Each one consists of a body and a ramus. The ramus of the mandible has the coronoid, condylar, and angular processes, as well as the masseteric fossa on its lateral surface. The coronoid process is the largest process of the three processes and it forms the dorsal part of the ramus and extends outward and upward. The condylar process articulates with the skull forming the temporomandibular joint. The angular process is medially directed. The mandible houses the dental alveoli for four incisors, a canine, three premolars, and four molars. There are mandibular foramina on the two rami of the mandible, near the angular processes, while the two mental foramina are present on each side of the body of the mandible laterally (Figure 1, Figure 3 and Figure 7).

## 4. Discussion

There is not much available literature reporting on the osteological description of the skull of the common opossum from the Caribbean. However, the skull of the white-eared opossum in South America, the short-tailed opossum, and the marsupial *Marmosa murina* are described [6,7,8], respectively. Most of the researches were done in the allometry of the skull of the opossum. The ontogeny of the skull was studied in the white-eared opossum and in the shrew opossums respectively [11,12].

The bones of the skull of the male and female common opossum are identical and are similar to that of the white-eared opossum, the short-tailed opossum, and the marsupial *Marmosa murina* [6,7,8], respectively. However, the jugal bone is absent in white-eared opossum [6]. On the other hand, the skull in the present study is homologues to the skull of other animals, such as in the dog and in the ferret (*Mustela putoris furo*), in the porcupine (*Hystrix cristata*), and in the Koala (*Phascolarctos cinereus*), respectively [13,14,15,16].

The present results as well as that in the white-eared opossum [6], in the short-tailed opossum [7], in the marsupial *Marmosa murina* [8], and in the Koala [15] stated that the skull is divided into short cranial and elongated facial regions, while the ferret has short facial region [13,16].

The supraorbital foramen is absent in the skull of the common opossum; a similar result is noticed in the white-eared opossum, in the raccoon, in the otter (*Lutra lutra*), in the badger, and in the marten skull [6,14,17,18,19]. On the other hand, the supraorbital foramen is present in domestic animals, such as the donkey (*Equius asinus*) and the sheep [20,21]. However, there is a foramen for the frontal diploic vein in the short-tailed opossum (*Monodelphis brevicaudata*) [7].

Similar to the observations in the dog [2], in the white-eared opossum [6], in the short-ailed opossum [7], and in the ferret [13], the orbit of the common opossum lacks of bony roof as it has an incomplete rim and the frontal process of the zygomatic bone to the zygomatic process of the frontal bone are completed by a ligament. Moreover, the bony orbit of the common opossum is formed by the frontal, lacrimal bone, zygomatic bone, maxillary bone, palatine bone, and squamosal bone, as well as the basisphenoid and alisphenoid of the sphenoid bone. Similar findings are reported in the short-tailed opossum [7], in the marsupial *Marmosa murina* [8], and in the Koala [15,22]. The lack of bony roof of the orbit in the common opossum could be to facilitate the wide opening of the jaws.

The external sagittal crest on the dorsal aspect of the skull of the common opossum is prominent; similar result is recorded in the white-eared opossum [6], in the short-tailed opossum [7], in the ferret [13], and in the European badger [18] On the other hand, the external sagittal crest is less prominent in the Koala [15]. Moreover, the prominent external sagittal crest gives enough area for the attachment of the temporalis muscle, which is one of the main chewing muscles.

The zygomatic arch of the common opossum is formed by the temporal process of the zygomatic bone as well as the zygomatic processes of the maxilla and squamosal bones; similar result is noticed in the white-eared opossum [6], in the short-tailed opossum [7], and in the marsupial *Marmosa murina* [8], while the zygomatic arch is formed by the squamous temporal and zygomatic bone in the dog [2]. However, the zygomatic arch is formed by the zygomatic process of the maxilla and the zygomatic process of the temporal bone in ferret [13]. Moreover, the zygomatic arch acts as the origin of the masseter muscle, supports the eye, and provides surface in the articulation of the mandible [13].

The tympanic bulla is absent in the common opossum while there is the tympanic process of the alisphenoid; similar result is observed in the short-tailed opossum [7] and in the marsupial *Marmosa murina* [8], while the tympanic bulla is ill-developed in the white-eared opossum [6]. Meanwhile, the tympanic bulla is well developed in the dog [2,19], in the ferret [13], and in the porcupine [14]. The osseous auditory bulla shows significant ventral expansion in the koala [15].

The bony part of the hard palate in the common opossum is similar to that in the dog [2] and in the white-eared opossum [6]. The hard palate has foramina and fenestrae, which vary in occurrence, size, and position among marsupials [8].

Concerning the nasal septum, the present study reveals that the nasal cavity of the common opossum is divided into right and left halves by the nasal septum, which is cartilaginous in its rostral third and osseous in its caudal two thirds. Similar findings are noticed in the short-tailed opossum [7], in the marsupial *Marmosa murina* [8], and in the koala [15,23].

The present investigation records that the dental formula in the common opossum corresponds to that of the other opossum such as the white-eared opossum [5], and the marsupial *Marmosa murina* [8], but it is different to that of the Koala [16].

Most of the foramina in the skull of the common opossum are similar with those that are described in the white-eared opossum [6], in the short-tailed opossum [7], and in the marsupial *Marmosa murina* [8]. Moreover, the skull in the present study is similar to other mammals except that the maxillopalatine fenestra and the palatal foramen in the common opossum are the major palatine foramen and the palatine canal (minor palatine foramen), respectively, in domestic animals [6,7]. The minor palatine foramen transmits the minor palatine artery of the maxillary artery to the palate in the marsupials [24].

The bones of the cranium of the common opossum are the ethmoid, parietal, sphenoid, frontal and squamosal bones. Similar result is observed in in the white-eared opossum [6], in the short-tailed opossum [7], in the marsupial *Marmosa murina* [8], and in the Koala [15].

The mandible is the largest bone of the skull of the common opossum; similar result is noticed in the white-eared opossum [6], in the short-tailed opossum [7], in the marsupial *Marmosa murina* [8], and in the guinea pig [16]. Furthermore, the mandible has two parts that are firmly connected by at the mandibular symphysis, as mentioned in the white-eared opossum [6] and in the ferret [13].

Knowledge of the detailed description of the osteology and the foramina of the skull and mandible will help the veterinary surgeons and radiologist in the diagnosis and treatment of the head affections of the common opossum, especially since some people in Trinidad started to rear this animal for meat production [25].

## 5. Conclusions

The bones and foramina of the skull and mandible of the common opossum were described via the dorsal, ventral, and lateral views, as well as sagittal section of the skull and lateral and caudal views of the mandible. The present study revealed that there was no significant difference between the osteology and foramina of the skull and mandible of the common opossum from the Caribbean island of Trinidad and Tobago when compared with those described in other marsupials and/or other mammals. Moreover, this study can lead to future functional and comparative anatomical and radiological studies
